# Gas6/TAM system as potential biomarker for multiple sclerosis prognosis

**DOI:** 10.3389/fimmu.2024.1362960

**Published:** 2024-04-30

**Authors:** Davide D’Onghia, Donato Colangelo, Mattia Bellan, Stelvio Tonello, Chiara Puricelli, Eleonora Virgilio, Daria Apostolo, Rosalba Minisini, Luciana L. Ferreira, Leonardo Sozzi, Federica Vincenzi, Roberto Cantello, Cristoforo Comi, Mario Pirisi, Domizia Vecchio, Pier Paolo Sainaghi

**Affiliations:** ^1^ Department of Translational Medicine, University of Piemonte Orientale (UPO), Novara, Italy; ^2^ Center for Autoimmune and Allergic Diseases (CAAD), University of Piemonte Orientale (UPO), Novara, Italy; ^3^ Department of Health Sciences, Pharmacology, University of Piemonte Orientale (UPO), Novara, Italy; ^4^ Department of Health Sciences, Interdisciplinary Research Center of Autoimmune Diseases (IRCAD), University of Piemonte Orientale (UPO), Novara, Italy; ^5^ Internal Medicine and Rheumatology Unit, Azienda Ospedaliera Universitaria (AOU) “Maggiore della Carita”, Novara, Italy; ^6^ Department of Health Sciences, Clinical Biochemistry, University of Piemonte Orientale (UPO), Novara, Italy; ^7^ Department of Translational Medicine, Neurology Unit, Maggiore Della Carità Hospital, University of Piemonte Orientale, Novara, Italy; ^8^ Department of Translational Medicine, Neurology Unit, S. Andrea Hospital, University of Piemonte Orientale (UPO), Vercelli, Italy

**Keywords:** Gas6, TAM receptors, multiple sclerosis, inflammation, biomarker

## Abstract

**Introduction:**

The protein growth arrest-specific 6 (Gas6) and its tyrosine kinase receptors Tyro-3, Axl, and Mer (TAM) are ubiquitous proteins involved in regulating inflammation and apoptotic body clearance. Multiple sclerosis (MS) is the most common inflammatory demyelinating disease of the central nervous system leading to progressive and irreversible disability if not diagnosed and treated promptly. Gas6 and TAM receptors have been associated with neuronal remyelination and stimulation of oligodendrocyte survival. However, few data are available regarding clinical correlation in MS patients. We aimed to evaluate soluble levels of these molecules in the cerebrospinal fluid (CSF) and serum at MS diagnosis and correlate them with short-term disease severity.

**Methods:**

In a prospective cohort study, we enrolled 64 patients with a diagnosis of clinical isolated syndrome (CIS), radiological isolated syndrome (RIS) and relapsing–remitting (RR) MS according to the McDonald 2017 Criteria. Before any treatment initiation, we sampled the serum and CSF, and collected clinical data: disease course, presence of gadolinium-enhancing lesions, and expanded disability status score (EDSS). At the last clinical follow-up, we assessed EDSS and calculated MS severity score (MSSS) and age-related MS severity (ARMSS). Gas6 and TAM receptors were determined using an ELISA kit (R&D Systems) and compared to neurofilament (NFLs) levels evaluated with SimplePlex™ fluorescence-based immunoassay.

**Results:**

At diagnosis, serum sAxl was higher in patients receiving none or low-efficacy disease-modifying treatments (DMTs) *versus* patients with high-efficacy DMTs (*p* = 0.04). Higher CSF Gas6 and serum sAXL were associated with an EDSS <3 at diagnosis (*p* = 0.04; *p* = 0.037). Serum Gas6 correlates to a lower MSSS (r^2^ = −0.32, *p* = 0.01). Serum and CSF NFLs were confirmed as disability biomarkers in our cohort according to EDSS (*p* = 0.005; *p* = 0.002) and MSSS (r^2^ = 0.27, *p* = 0.03; r^2^ = 0.39, *p* = 0.001). Results were corroborated using multivariate analysis.

**Conclusions:**

Our data suggest a protective role of Gas6 and its receptors in patients with MS and suitable severity disease biomarkers.

## Introduction

Multiple sclerosis (MS) is an inflammatory demyelinating disease of the central nervous system (CNS) characterized by progressive and irreversible disability with a high impact on patients’ quality of life (1). Both inflammatory and neurodegenerative aspects contribute to the disease (2). Inflammation contributes to myelin destruction in the CNS, to damaging of oligodendrocytes (ODs), and activation of astrocytes with neuronal damage (3). It has also been observed that ODs undergo cell death in newly formed lesions, which preludes the appearance of extensive regions of demyelination (4). Growth arrest-specific 6 (Gas6) and its receptors have been shown to play a critical role in innate immune system homeostasis by regulating apoptosis and inflammation (5). Gas6 is a soluble glycoprotein of 75 kDa that belongs to the vitamin K-dependent protein family (6). To exert its biological functions, Gas6 must interact with a specific family of tyrosine kinase receptors, called TAM, consisting of three different receptors: Tyro-3, Axl, and Mer (7). TAM receptors play important roles in cell survival, growth, aggregation and migration, angiogenesis, and control of inflammatory responses, apoptotic cell and membrane engulfment, and phagocytic elimination (8). TAM receptors can be cleaved into their soluble forms (sTyro-3, sAxl, and sMer) by specific proteases, ADAM 10 and 17 (9). These soluble receptors can still bind Gas6 protein retaining their functions in the modulation of inflammation (7). TAM receptors are widely expressed in the nervous system, including ODs, and Gas6/TAMs have been associated with stimulation of OD survival and neuronal remyelination (10). Axl has the highest affinity for Gas6 (11), and the experimental evidence supports a direct role in neuronal myelinization (12). However, few data are available regarding the clinical correlation in MS patients. We aimed to assess the soluble levels of these molecules in the cerebrospinal fluid (CSF) and serum, at the time of MS diagnosis, and evaluate their possible correlations with short-term disease severity.

## Materials and methods

### Patients

In this observational prospective cohort study, we recruited, between October 2017 and February 2022, 64 patients (43 females) in “Maggiore della Carità” Hospital in Novara, Italy. All patients had a follow-up visit at least 1 year after their diagnosis, between July 2021 and December 2022. The clinical data were acquired twice, both at the diagnosis and at the last follow-up visit. CSF and serum samples were obtained at diagnosis while the patients underwent MS diagnostic work-up. The study’s inclusion criteria were the diagnosis of clinical isolated syndrome (CIS), radiological isolated syndrome (RIS), or relapsing–remitting (RR), according to McDonald 2017 (13) at the end of the follow-up.

### Ethical committee

All the participants signed an informed consent form. The study protocol was approved by the local Ethical Committee (CE 262/2022) and was conducted in accordance with the Declaration of Helsinki.

### Clinical evaluation

Demographic and clinical variables collected at diagnosis were sex, age at onset, clinical course, the presence of gadolinium-enhancing (Gad+) lesions, and disability according to the expanded disability status score (EDSS) (14). Brain and spinal imaging were performed within 3 months from the diagnosis on a 1.5-Tesla MRI with a single dose of Gad. EDSS was used to assess disability and monitor changes over time. This score has been corrected by time-measure using the *MS* severity score (MSSS) (15) and by the age using the age-related MS severity (ARMSS) (16).

### Sample collection and biomarker determinations

Cerebrospinal fluid (CSF) was collected through lumbar puncture at diagnosis. CSF was centrifuged at 1,300 rpm for 10 min and stored at −80°C until the analysis. At the time of CSF collection, all patients were treatment naïve (including disease-modifying treatments or DMTs and steroids). Serum was immediately collected by centrifugation at 3,500 rpm for 15 min and stored at −80°C until the analysis time. CSF and serum NFLs were measured with the Simple PlexTM fluorescence-based immunoassay by Bio-Techne with the Ella SimplePlex™ Platform (Bio-Techne s.r.l., Milan, Italy). NFLs were measured using the Human NFL SimplePlex™ Cartridge Kit (Lot no. 21519). All kit components (cartridge, sample diluent SD13, and Wash Buffer A) were provided ready to use, and they were allowed to reach room temperature before use. CSF and serum levels of Gas6 were determined with ELISA technique using a commercial kit (R&D Systems Duo Set Elisa DY6488, McKinley, MN, USA) and following the manufacturer’s instructions. Samples were diluted 1:50 in a sample diluent. The optical density at 450 nm was fitted versus a calibration curve prepared with a standard (0–1 ng/ml range), as suggested by the manufacturer. CSF and serum levels of sTyro-3 were determined with the commercially available T ELISA kit (R&D Systems Duo Set Elisa DY6488, McKinley, MN, USA) following the manufacturer’s instructions. Samples were diluted 1:5 in a sample diluent. The optical density at 450 nm was fitted versus a calibration curve prepared with a standard (0–4 ng/ml range), as suggested by the manufacturer. The ELISA technique determined CSF and serum levels of sAxl by using a commercial kit (R&D Systems Duo Set Elisa DY6488, McKinley, MN, USA) and following the manufacturer’s instructions. Samples were diluted 1:25 in a sample diluent. The optical density at 450 nm was fitted versus a calibration curve prepared with a standard (0–4 ng/ml range), as suggested by the manufacturer. The ELISA technique determined CSF and serum levels of sMer using a commercial kit (R&D Systems Duo Set Elisa DY6488, McKinley, MN, USA) and following the manufacturer’s instructions. Samples were diluted 1:2 in a sample diluent. The optical density at 450 nm was fitted versus a calibration curve prepared with a standard (0–10 ng/ml range), as suggested by the manufacturer. Absorbance was recorded using a Victor X4 microplate reader (Perkin Elmer, Waltham, MA, USA).

### Statistical analysis

For continuous variables, the measures of centrality and dispersion were medians and interquartile ranges [IQR], and comparisons between groups regarding these variables were performed using the Mann–Whitney U-test and the Kruskal–Wallis test. The Pearson χ2 was used to analyze the association between categorical variables shown as frequencies (%). Correlations were performed with Spearman’s rank correlation coefficient and linear regression for significant predictors in the univariate model. Multivariable regressions were built to identify the variables independently associated with the severity score. The threshold for statistical significance was 0.05 (two tailed). Statistical analyses were performed with Stata statistical software version 17.0 (Stata Corp, 4905 Lakeway Drive College Station, TX, USA), while graphs were created using GraphPad Prism version 9.4.0 (GraphPad Software, La Jolla, CA, USA).

## Results

The main features of our 64 patients are reported in **Table 1**.

**Table 1:** General features of the study population and their clinical parameters.

Demographics parameters and clinical scores	# of patients
Sex (F/M)	43 (67.19)/21 (32.81)
Age (years)	37 [19.0–61.0]
Age at onset (years)	32 [14.0–56.0]
Disease course	
Radiological isolated syndrome	2 (3.12)
Clinical isolated syndrome	3 (4.69)
Relapsing–remitting MS	59 (92.19)
MRI features	
Gadolinium-enhancing lesions	39 (60.94)
Brain lesions >10	36 (56.25)
Spinal lesion (yes)	44 (67.19)
Disability measures	
Switch from first disease-modifying treatments within 1 year	9 (5.7)*
EDSS at diagnosis EDSS < 3 at diagnosis	1.5 [0.0–6.0] 55 (85.94)
EDSS at last follow-up EDSS < 3 at last follow-up	1.5 [0.0–6.5] 56 (87.5)
MSSS at last follow-up	2.85 [0.24–9.59]
ARMSS at last follow-up	3.22 [0.29–8.47]
Biomarkers at diagnosis	
**NFLs (pg/ml)** Serum CSF	29.55 [12.1–262] 1,590.5 [201–35,824]
**Gas 6 (ng/ml)** Serum CSF	23.49 [12.26–54.65] 7.76 [1.80–32.75]
**sAxl (ng/ml)** Serum CSF	29.22 [15.42–231.3] 26.38 [7.9–48.19]
**sMer (ng/ml)** Serum CSF	2.54 [0.0–55.1] 0.0 [0.0–0.0]
**sTyro-3 (ng/ml)** Serum CSF	3.54 [1.77–9.63] 3.79 [1.71–6.56]

Continuous variables are presented as medians [IQR] and categorical variables as frequencies (%). CSF, cerebrospinal fluid; OB, oligoclonal bands; EDSS, expanded disability status scale, MSSS, multiple sclerosis severity score; ARMSS, age-related multiple sclerosis severity; NFLs, neurofilaments.

*Of the patients, 3/9 stopped/changed the first DMT for side effects, not for efficacy.

**Figure 1:**
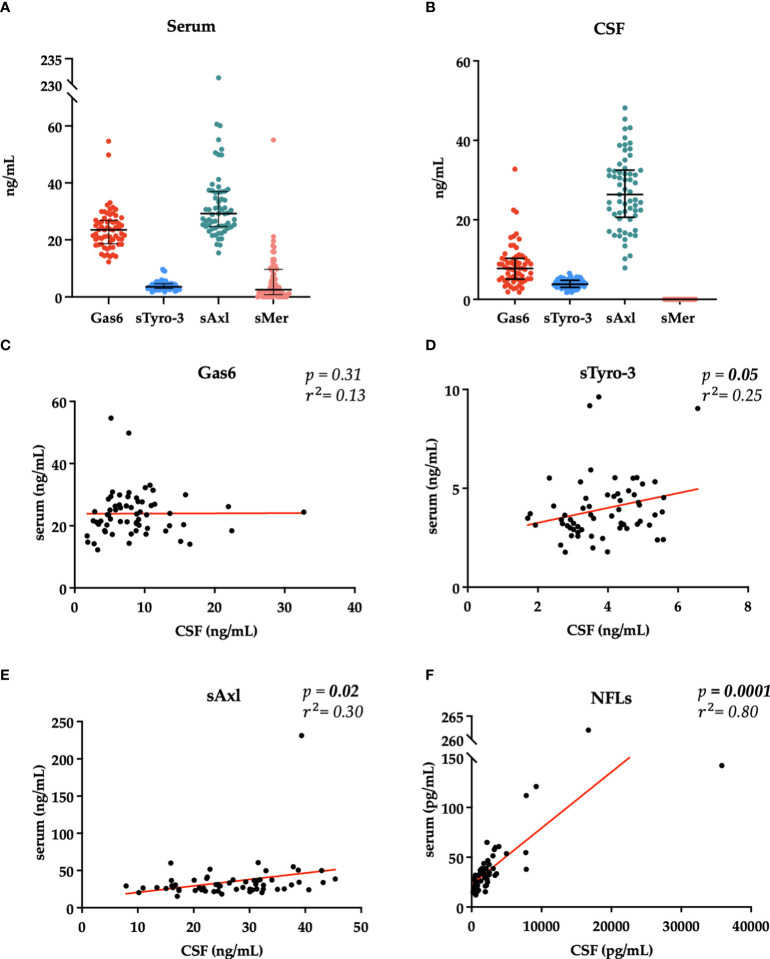
Distribution of Gas6 and TAM receptor concentrations in serum **(A)** and CSF **(B)**. Results are shown as medians [IQR]. Spearman’s rank correlation between serum and CSF levels of Gas6 **(C)**, sTyro-3 **(D)**, sAxl (**(E)**, and NFLs **(F)** concentrations. r^2^, coefficient of correlation; p, p-value.

Initially, we investigated serum and CSF levels of Gas6 and its receptor. All molecules were detectable except for CSF sMer. Gas6, sAxl, and sMer concentrations resulted moderately higher in the serum than in the CSF, thus showing an opposite trend to NFLs levels that are more elevated in the CSF as largely reported (17). Our data show no statistically significant correlation between serum and CSF concentrations of Gas6, whereas serum and CSF levels of sTyro-3 (p = 0.05), sAxl (p = 0.02), and NFLs (p = 0.0001) were significantly related between the two fluids (sMer was not analyzed since it was undetectable in the CSF) (**Figure 1**).

We compared the RIS–CIS population to those patients with RRMS and found higher sMer and sTyro-3 serum levels at the diagnosis in the RIS-CIS subgroup (**Supplementary Figure 1**). No statistically significant results were found in the CSF.

### MS treatments and disability

**Figure 2:**
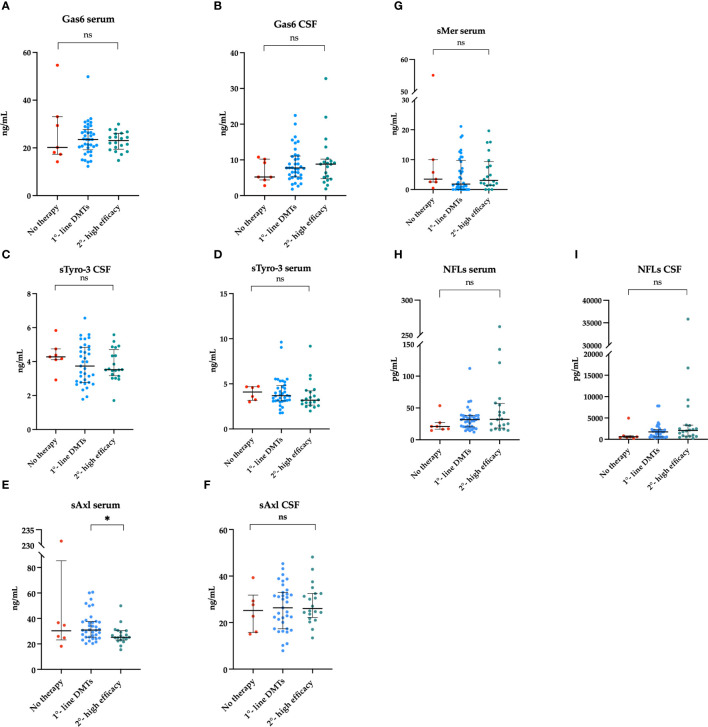
Association between Gas6 concentration in the serum **(A)** and CSF **(B)**, sTyro3 concentration in the serum **(C)** and CSF **(D)**, sAxl concentration in the serum **(E)** and CSF **(F)**, sMer concentration in the serum **(G)**, NFLs concentration in the serum **(H)** and CSF **(I)** and the type of therapy at follow-up visit. Results are shown as medians [IQR]. *p = 0.04, ns, not significant.

At the end of the follow-up, 7 (11%) patients were receiving no treatment; 37 (58%), a low-efficacy; and 20 (31%), high-efficacy DMTs. Six (9%) patients switched to high-efficacy therapy during the follow up. Instead, 3 (5%) patients stopped/changed the first DMTs for side effects (not for inefficacy). As shown in **Figure 2**, serum sAxl was higher in those patients who underwent no treatment or on low-efficacy DMTs.

**Figure 3:**
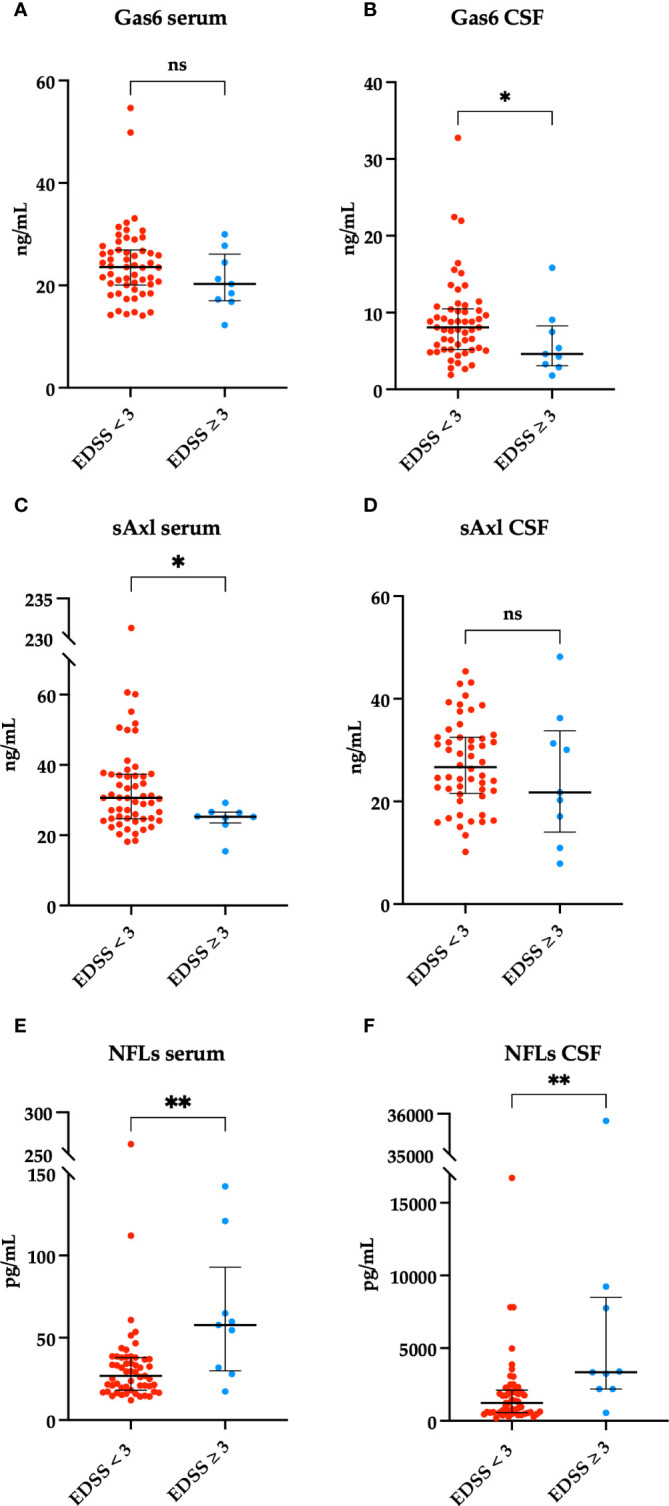
Associations between Gas6 levels in the serum and CSF (ng/ml) and < 3 or ≥ 3 EDSS clinical scores on the first visit. *p = 0.04 **(A, B)**. sAxl levels in the serum and CSF (ng/ml) in patients with < 3 or ≥ 3 EDSS clinical scores on the first visit. *p = 0.037 **(C, D)**. Associations between NFLs levels in the serum and CSF (pg/mL) and < 3 or ≥ 3 EDSS clinical scores on the first visit. **p = 0.005, **p = 0.002 **(E, F)**. Results are shown as medians [IQR]. ns, not significant.

**Figure 4:**
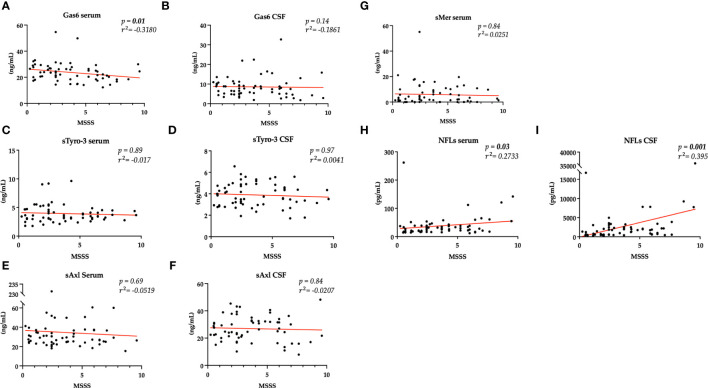
Spearman’s correlation between MSSS and Gas6 levels in the serum and CSF **(A, B)**, TAM receptors levels in the serum and CSF **(C–G)**, and NFLs levels in the serum and CSF **(H, I)**. r^2^ = coefficient of correlation, p = p-value.

To evaluate disability measures, we divided our patients according to EDSS scores < 3 or ≥ 3 at first and follow-up visit. As shown in **Figure 3**, we found a higher serum sAxl and CSF Gas6 levels in those patients with EDSS < 3 at diagnosis. As expected, higher NFL levels in the CSF and serum were associated with higher EDSS scores at diagnosis. No significant result associations were observed with the EDSS at follow-up visit (data not shown). Subsequently, we considered disability according to MSSS and ARMSS at the last follow-up. As shown in **Figure 4**, an inverse correlation was found only for serum Gas6 and MSSS. On the other hand, as expected, NFL levels in the serum and CSF directly correlated with MSSS. No other significant correlations were found with ARMSS (**Supplementary Figure 2**).

We did not find any difference in Gas6 and TAM receptors according to the number of brain, spinal, and gadolinium-enhancing lesions (**Supplementary Table 2**).

### Multivariate analysis

We finally performed multivariate regression models to predict MS disability according to EDSS at diagnosis (**Table 2**) and MSSS at last follow-up (**Table 3**). The included independent variables were gender, age, number of lesions, and the other serum biomarkers. We did not find statistically significative predictors in the multivariate analyses for different types of MS and in the type of therapy at follow- up visit (**Supplementary Tables 8, 9**).

**Table 2:** Multivariate regression model of EDSS < 3 at diagnosis including demographic and severity variables.

Predictor	Coefficient	Standard error	p*-*value	95% confidence interval
Gas6 serum (ng/mL)	−0.0169	0.0075	**0.029**	−0.0320–−0.0018
Gas6 CSF (ng/mL)	−0.0192	0.0089	**0.037**	−0.0372–−0.0012
sAXL serum (ng/mL)	−0.0026	0.0044	0.55	−0.0112–0.0063
sMer serum (ng/mL)	0.0067	0.0081	0.41	−0.0097–0.0231
sTyro-3 serum (ng/mL)	0.0002	0.0290	0.99	−0.0582–0.0586
NFL serum (pg/mL)	0.0020	0.0011	0.21	−0.0026–0.0044
Age	0.0025	0.0045	0.58	−0.0066–0.0116
Gender	−0.060	0.0936	0.52	−0.2487–0.1279
N° brain lesion >10	0.1444	0.0907	0.11	−0.0381–0.3269
Spinal lesion	0.0985	0.0900	0.28	0.0819–0.2871
Gadolinium enhancing	0.7653	0.6075	0.41	−0.0826–0.2796

In bold are indicated statistically significant results (*p* < 0.05). After the multivariate analysis, only serum and CSF Gas6 levels (p = 0.029; p = 0.037) resulted as predictors for the disability at the first visit according to EDSS.

**Table 3:** Multivariate regression model of MSSS at last follow-up including demographic and severity variables.

Predictor	Coefficient	Standard error	p*-*value	95% confidence interval
Gas6 serum (ng/mL)	−0.1577	0.0503	**0.003**	−0.2588–−0.0565
sAXL serum (ng/mL)	0.0317	0.0285	0.27	−0.0256–0.1227
sMer serum (ng/mL)	0.0192	0.0515	0.71	−0.0843–0.1205
sTyro-3 serum (ng/mL)	0.0687	0.1983	0.73	−0.3298–0.4673
NFL serum (pg/mL)	0.0098	0.0078	0.21	−0.0059–0.0257
Age	0.0688	0.0305	**0.029**	0.0074–0.1302
Gender	−0.7116	0.6308	0.26	−1.9793–0.5560
N° brain lesion >10	0.2788	0.5978	0.64	−0.9226–1.4803
Spinal lesion	1.3529	0.6075	**0.035**	0.0970–2.6088
Gadolinium enhancing	0.7653	0.6075	0.41	−0.4555–1.9861

In bold are indicated statistically significant results (*p* < 0.05).Disability over time according to MSSS was predicted by serum Gas6 (p = 0.003), age, and the presence of spinal lesions.

## Discussion

In our prospective cohort study, first, we evaluated the CSF and serum Gas6 and TAM receptor levels in MS patients at diagnosis. All biomarkers were detectable, except for sMer that was absent in the CSF, as also previously reported and discussed by our group (18). The absence of sMer could be related to a lower expression in the brain compared to sAxl and sTyro-3 (19). Moreover, in the present study, we first compared Gas6 and TAM receptors to NFLs: Gas6, sAxl, and sMer levels resulted higher in the serum than in the CSF, thus showing an opposite trend to that of NFLs (17). A possible role of TAM receptors in MS is related to the clearance of myelin debris for the remyelination process, which can be reduced by ineffective phagocytosis (20), as could happen in the dysregulation of TAM signaling (21). Among the TAM receptors, Tyro-3 could be the main actor in mediating the promyelinating effects of Gas6 during developmental myelination (10). Consequently, loss of Tyro-3 causes a delay in myelinization and a reduction in myelin thickness both *in vitro* and *in vivo* (22, 23). Looking at Mer and Axl, they regulate microglial functions (24, 25) and normally drive phagocytosis of apoptotic cells generated during adult neurogenesis (26, 27).

Second, we searched for any association with clinical features at disease diagnosis. Looking at disability at diagnosis, those patients with EDSS score < 3 showed higher levels of CSF Gas6 and serum sAxl levels, whereas, as expected, there was a statistically significant correlation between higher levels of CSF and serum NFLs and EDSS ≥ 3 (28). Our data suggested a role of sAxl to identify those cases with low disability at onset and then treated with low-efficacy DMTs. A possible pathogenic hypothesis is related to the Gas6 and TAM receptors expression in several cell types in the nervous system, including ODs (29). Activation of the Axl receptor by Gas6 induces an intracellular response that promotes oligodendrocyte survival and stimulates the myelination process (30). Nonetheless, hyperactivation of the immune system also contributes to impaired remyelination, as demonstrated in experimental autoimmune encephalomyelitis. In this mouse model, loss of Axl increases central nervous system inflammation delaying the removal of myelin debris (12). Furthermore, several studies in Gas6 and Axl-knockout mice showed remyelination abnormalities due to increased microglia activation confirming specific contributions of Gas6/Axl signaling in the remyelination processes. Exposure to toxic cuprizone resulted in axon damage in mutant mice, which is associated with an abnormal inflammatory response due to reduced SOCS expression, suggesting that Gas6/Axl signaling may be important in reducing CNS inflammation and maintaining axon integrity after demyelinating/proinflammatory stimuli (12, 31–35).

Third, a prognostic role over time emerged only for serum Gas6 since lower levels of this biomarker is related to higher MSSS. This result suggests a protective role in MS. As expected, on the contrary, higher CSF and serum NFLs levels are related to higher MSSS score. Gas6 is involved in different cellular processes with anti-inflammatory, neuroprotective, promyelinating properties, and a biomarker for acute disease course. On the other side, our group measured CSF and plasma Gas6 protein during relapses in relation to the clinical features (symptoms) and severity scores as the Kurtzke Functional System (FS) showing the usefulness of Gas6 as a biomarker of an acute disease course (36–39).

Moreover, the Gas6 TAM pathway is involved in viral response, including thus EBV infection (2, 40–42), increasing during a viral infection (7). Gas6 may act as a modulator of inflammation, regulating the immune response and limiting the inflammation and tissue damage associated with viral infection (43). Furthermore, activation of TAM receptors by Gas6 may influence the response of immune cells, including macrophages and dendritic cells, by promoting phagocytosis of infected cells and antigen presentation (44, 45). Viral infection can also influence TAM receptors expression and Gas6 production (46). For instance, during EBV infection, it has been observed that Axl expression can increase in infected cells (47). However, the direct link between Gas6 and EBV still needs several studies to be proven.

With the present work, we focused our attention on prognosis and disability using different clinical scores that better indicate a disease course, such as the MSSS and the ARMSS. Although our study is a pilot analysis with some limitations, like the number of patients involved and the monocentric nature of the recruitment, results are promising and could be extended by the Gas6/TAM levels follow-up during the entire evolution of the pathology.

In conclusion, the Gas6-TAM axis showed a trend to identify those patients that could be considered more “benign.” In fact, serum sAxl was higher in those patients with lower disability at onset, and serum Gas6 was higher in patients with lower disability over time.

Our study suggests serum Gas6 as a reliable prognostic biomarker; however, prospective further investigation about the protective role of the Gas6/TAM system role is needed.

## Data availability statement

The original contributions presented in the study are included in the article/**Supplementary Material**. Further inquiries can be directed to the corresponding author.

## Ethics statement

The studies involving humans were approved by Ethical Committee (CE 262/2022). The studies were conducted in accordance with the local legislation and institutional requirements. The participants provided their written informed consent to participate in this study.

## Author contributions

DD: Conceptualization, Data curation, Formal analysis, Methodology, Visualization, Writing – original draft, Writing – review & editing. DC: Conceptualization, Data curation, Formal analysis, Investigation, Project administration, Supervision, Writing – original draft, Writing – review & editing. MB: Conceptualization, Data curation, Methodology, Supervision, Writing – original draft, Writing – review & editing, Formal analysis, Project administration. ST: Conceptualization, Data curation, Investigation, Methodology, Supervision, Writing – review & editing, Project administration. CP: Formal analysis, Methodology, Validation, Writing – review & editing. EV: Investigation, Conceptualization, Data curation, Methodology, Project administration, Supervision, Writing – review & editing. DA: Formal analysis, Data curation, Methodology, Writing – original draft, Writing – review & editing. RM: Conceptualization, Project administration, Supervision, Validation, Writing – review & editing. LF: Methodology, Conceptualization, Data curation, Formal analysis, Supervision, Writing – review & editing. LS: Methodology, Writing – review & editing. FV: Methodology, Writing – review & editing. RC: Writing – review & editing, Conceptualization, Data curation, Investigation, Project administration, Supervision, Validation, Visualization, Writing – original draft. CC: Conceptualization, Formal analysis, Investigation, Project administration, Supervision, Validation, Visualization, Writing – original draft, Writing – review & editing. MP: Conceptualization, Data curation, Formal analysis, Methodology, Supervision, Validation, Visualization, Writing – original draft, Writing – review & editing. DV: Conceptualization, Data curation, Formal analysis, Investigation, Methodology, Project administration, Supervision, Visualization, Writing – original draft, Writing – review & editing. PS: Conceptualization, Data curation, Formal analysis, Funding acquisition, Investigation, Methodology, Project administration, Resources, Supervision, Validation, Visualization, Writing – original draft, Writing – review & editing.
