# Supplementary material for: Gas6/TAM system as potential biomarker for multiple sclerosis prognosis
Source: Front Immunol. 2024 Apr 30;15:1362960. doi: 10.3389/fimmu.2024.1362960 (PMC11091300; doi:10.3389/fimmu.2024.1362960)
Supplement: Supplementary file 1 [file DataSheet_1.pdf]

Supplementary Materials.

Table 1. Correlation between serum and CSF concentrations of Gas6 and its receptor.

	Serum and CSF concentrations	
Variables	Correlation Coefficient	*p*-Value
Gas 6 (ng/mL)	0.13	0.31
sAXL (ng/mL)	0.30	**0.02**
sMER (ng/mL)	-	-

MS course and MRI features at diagnosis.

We compared the RIS-CIS population to those patients with RRMS and found higher sMer and sTyro-3 serum levels at the diagnosis in the RIS-CIS subgroup (**Figure 1**). No statistically significant results were found in CSF.

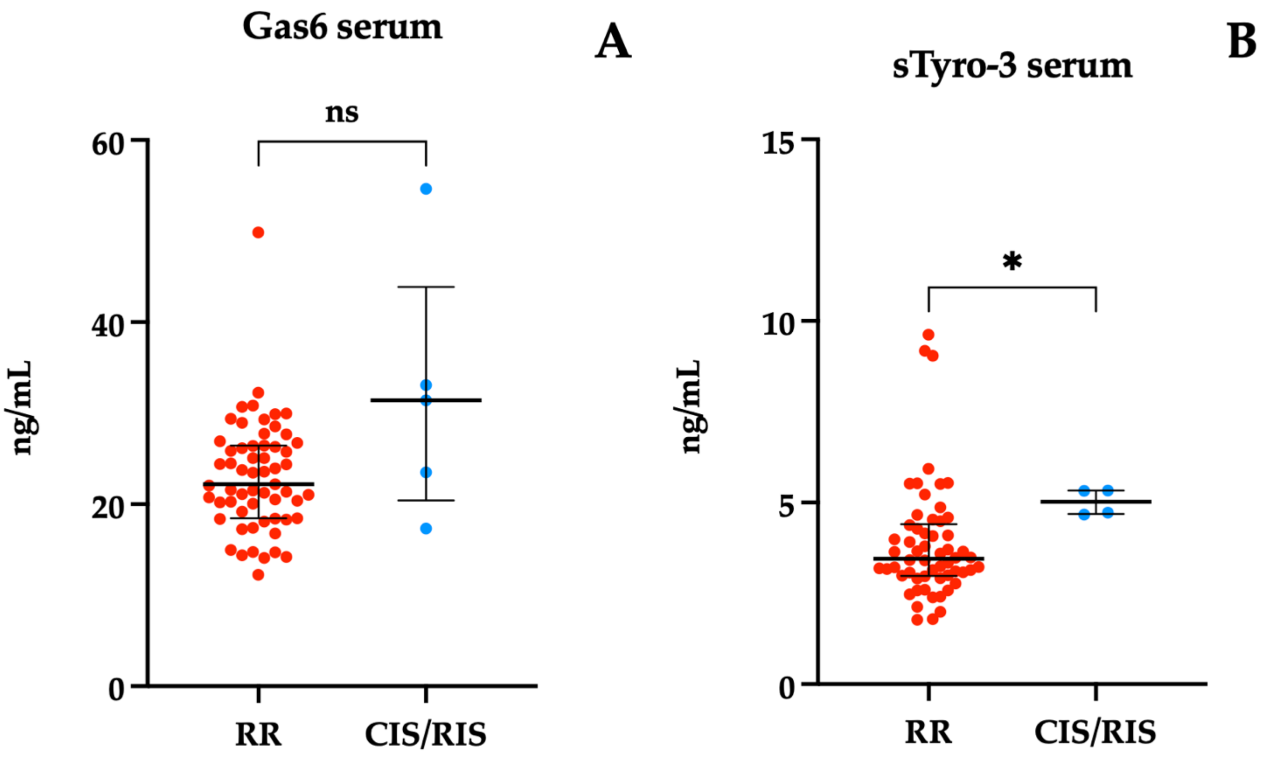

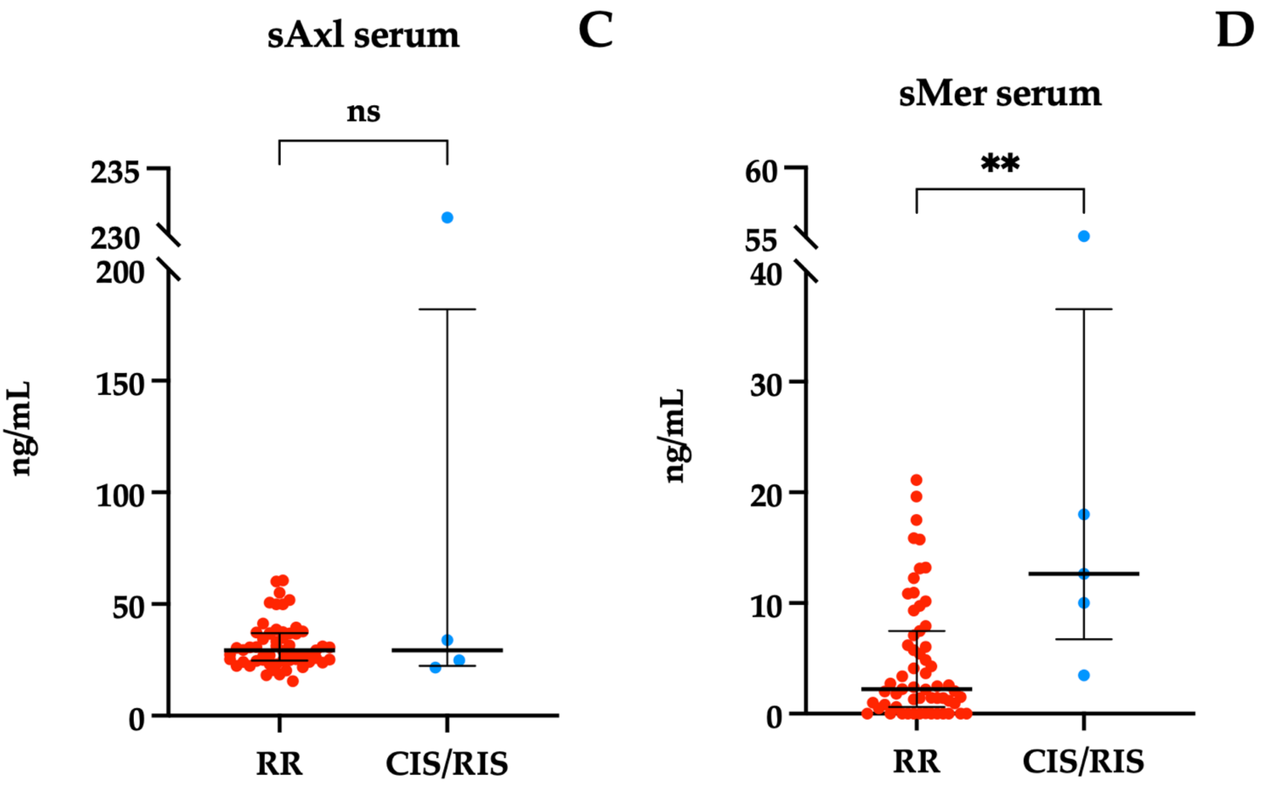

Figure 1. Association between Gas6 (**A**) and TAMs receptors (**B**, **C**, **D**) concentrations in serum with the different types of MS. RR = relapsing and remitting, RIS = Radiological Isolated Syndrome and CIS = Clinical Isolated Syndrome. Results are shown as medians [IQR]. * $p = 0.018$ and ** $p = 0.007$, ns = not significant.

Table 2. The table shows the associations between Gas6 and TAM receptor and T2 brain, spinal and gadolinium-enhancing lesions. Values are expressed as median [IQR]. N, number of patients.

	**Brain lesions <10 N = 28**	**Brain lesions >10 N = 36**		
**Variables**	**Median [IQR]**	**Median [IQR]**	**Z**	***p*-Value**
Gas 6 Serum (ng/mL)	22.84 [19.25 - 26.56]	23.71 [18.82 - 27.72]	-0.162	0.88
Gas6 CSF (ng/mL)	6.97 [4.97 - 10.24]	8.07 [5.05 - 10.41]	-0.436	0.67
sAXL Serum (ng/mL)	31.19 [24.59 - 37.72]	27.27 [24.77 – 33.64]	0.899	0.38
sAXL CSF (ng/mL)	22.91 [17.32 - 31.52]	29.70 [22.45 - 32.97]	-1.438	0.15
sMER Serum (ng/mL)	2.20 [0.01 – 7.53]	2.94 [1.45 – 10.89]	-1.392	0.17
sTyro-3 Serum (ng/mL)	1.72 [3.01 – 4.72]	1.29 [2.99 – 4.28]	0.62	0.54
sTyro-3 CSF (ng/mL)	1.62 [2.92 – 4.54]	1.90 [3.02 – 4.75]	-0.97	0.33

	Spinal lesions <10 N = 21	Spinal lesions >10 N = 43		
**Variables**	**Median [IQR]**	**Median [IQR]**	**Z**	***p*-Value**
Gas 6 Serum (ng/mL)	23.50 [18.45 – 29.89]	23.48 [19.20 - 26.39]	0.636	0.22
Gas6 CSF (ng/mL)	8.83 [6.43 - 10.10]	7.43 [4.82 - 10.94]	0.773	0.45
sAXL Serum (ng/mL)	30.03 [21.97 – 35.68]	29.04 [24.83 – 36.89]	-0.286	0.78
sAXL CSF (ng/mL)	23.64 [17.07 - 31.55]	26.71 [21.40 – 32.97]	-0.818	0.42
sMER Serum (ng/mL)	3.66 [1.19 - 10.01]	2.23 [0.81 – 7.47]	0.746	0.46
sTyro-3 Serum (ng/mL)	1.96 [3.06 – 5.02]	1.09 [2.98 – 4.08]	1.68	0.09
sTyro-3 CSF (ng/mL)	1.43 [3.28 – 4.72]	1.86 [2.96 – 4.82]	0.57	0.57

	**Gadolinium enhancing Lesion – N = 25**	**Gadolinium enhancing Lesion + N = 39**		
**Variables**	**Median [IQR]**	**Median [IQR]**	**Z**	***p*-Value**
Gas 6 Serum (ng/mL)	23.48 [18.32 - 26.39]	23.50 [20.05 – 27.68]	-0.117	0.91
Gas6 CSF (ng/mL)	7.39 [5.20 – 9.15]	8.27 [5.05 - 10.41]	-0.478	0.64
sAXL Serum (ng/mL)	30.68 [26.27 – 37.30]	26.83 [23.85 - 36.70]	1.561	0.12
sAXL CSF (ng/mL)	24.49 [18.87 - 32.19]	29.32 [21.76 – 32.52]	-0.850	0.40
sMER Serum (ng/mL)	3.47 [0.81 - 10.00]	2.50 [0.95 – 9.32]	0.207	0.84
sTyro-3 Serum (ng/mL)	1.67 [3.13 – 4.79]	1.31 [2.97 – 4.28]	0.74	0.46
sTyro-3 CSF (ng/mL)	1.69 [3.14 – 4.82]	1.43 [3.02 – 4.45]	0.60	0.55

Table 3. Association between biomarker and type of treatment at first visit

	No treatment	1° line	2° line		
Variables	Median [IQR]	Median [IQR]	Median [IQR]	χ^2	*p*-Value
Gas 6 Serum (ng/mL)	20.19 [17.32 – 33.07]	23.50 [20.05 – 26.92]	23.06 [19.73 – 26.01]	0.699	0.70
Gas6 CSF (ng/mL)	5.20 [4.38 – 10.20]	7.68 [5.23 – 10.61]	8.81 [4.86 – 10.03]	0.299	0.86
sAXL Serum (ng/mL)	30.31 [24.76 – 36.64]	31.11 [25.35 – 37.72]	25.19 [23.04 – 30.38]	4.790	0.09
sAXL CSF (ng/mL)	25.20 [16.01 – 29.32]	26.38 [17.35 – 32.97]	26.04 [22.70 – 32.51]	0.366	0.83
sMER Serum (ng/mL)	3.47 [2.50 – 10.01]	1.82 [0.03 – 9.32]	3.07 [1.46 – 8.84]	1.882	0.39

Table 4. Association between biomarker and type of treatment at follow – up

	No treatment	1° line	2° line		
Variables	Median [IQR]	Median [IQR]	Median [IQR]	χ^2	*p*-Value
Gas 6 Serum (ng/mL)	24.79 [17.32 – 33.07]	23.52 [20.05 – 26.92]	21.77 [18.82 – 25.18]	0.034	0.98
Gas6 CSF (ng/mL)	7.20 [5.18 – 10.20]	7.68 [5.12 – 10.94]	8.08 [4.28 – 9.38]	0.542	0.76
sAXL Serum (ng/mL)	34.71 [24.76 – 36.64]	30.70 [25.35 – 37.44]	25.27 [23.88 – 29.79]	6.285	**0.04**
sAXL CSF (ng/mL)	27.66 [22.75 – 29.32]	25.04 [17.35 – 32.24]	26.38 [21.76 – 32.52]	0.704	0.70
sMER Serum (ng/mL)	3.03 [2.50 – 10.01]	2.10 [0.03 – 9.32]	4.57 [1.46 – 8.84]	1.723	0.42

Table 5. We performed Mann Whitney U test between Gas6 and TAM receptor and the different clinical marker of severity of disease. Statistically significant results are evidenced in bold text.

	EDSS 1° visit < 3 N = 55	EDSS 1° visit > 3 N = 9		
Variables	Median [IQR]	Median [IQR]	Z	p-Value
Gas 6 Serum (ng/mL)	23.57 [20.05 – 26.92]	20.61 [17.26 – 24.50]	1.265	0.21
Gas6 CSF (ng/mL)	8.08 [5.20 – 10.41]	4.60 [3.29 – 7.48]	2.043	**0.04**
sAXL Serum (ng/mL)	30.63 [24.71 – 37.30]	25.27 [23.98 – 26.50]	2.079	**0.037**
sAXL CSF (ng/mL)	26.71 [21.75 – 32.50]	21.76 [17.07 – 31.31]	0.844	0.41
sMER Serum (ng/mL)	2.50 [0.61 – 9.32]	2.74 [1.30 – 9.76]	-0.058	0.96

	EDSS 2° visit < 3 N = 57	EDSS 2° visit > 3 N = 9		
Variables	Median [IQR]	Median [IQR]	Z	p-Value
Gas 6 Serum (ng/mL)	23.50 [20.05 – 26.92]	20.26 [18.31 – 26.15]	0.656	0.53
Gas6 CSF (ng/mL)	7.78 [5.19 – 10.15]	4.60 [2.88 – 15.84]	0.481	0.64
sAXL Serum (ng/mL)	29.33 [24.65 – 36.79]	27.82 [24.93 – 49.94]	-0.071	0.95
sAXL CSF (ng/mL)	26.72 [21.40 – 32.48]	21.76 [15.90 – 42.92]	0.577	0.57
sMER Serum (ng/mL)	2.41 [0.81 – 9.32]	3.39 [1.42 – 9.76]	-0.367	0.72

Table 6. We performed Spearman's correlation between Gas6 and TAM receptor and MSSS. Statistically significant results are evidenced in bold text.

	MSSS	
Laboratory Parameters	Correlation Coefficient	p-Value
Gas 6 Serum (ng/mL)	-0.3180	**0.01**
Gas6 CSF (ng/mL)	-0.1861	0.14
sAXL Serum (ng/mL)	-0.0519	0.69
sAXL CSF (ng/mL)	-0.0207	0.87
sMER Serum (ng/mL)	0.0251	0.84

Table 7. We performed Spearman's correlation between Gas6 and TAM receptor and ARMSS.

	ARMSS	
Laboratory Parameters	Correlation Coefficient	p-Value
Gas 6 Serum (ng/mL)	-0.2146	0.08
Gas6 CSF (ng/mL)	0.0436	0.73
sAXL Serum (ng/mL)	0.0142	0.91
sAXL CSF (ng/mL)	0.0473	0.72
sMER Serum (ng/mL)	-0.0386	0.76

In **Figure 2** are shown the Spearman's correlation between Gas6 and TAM receptor and ARMSS.

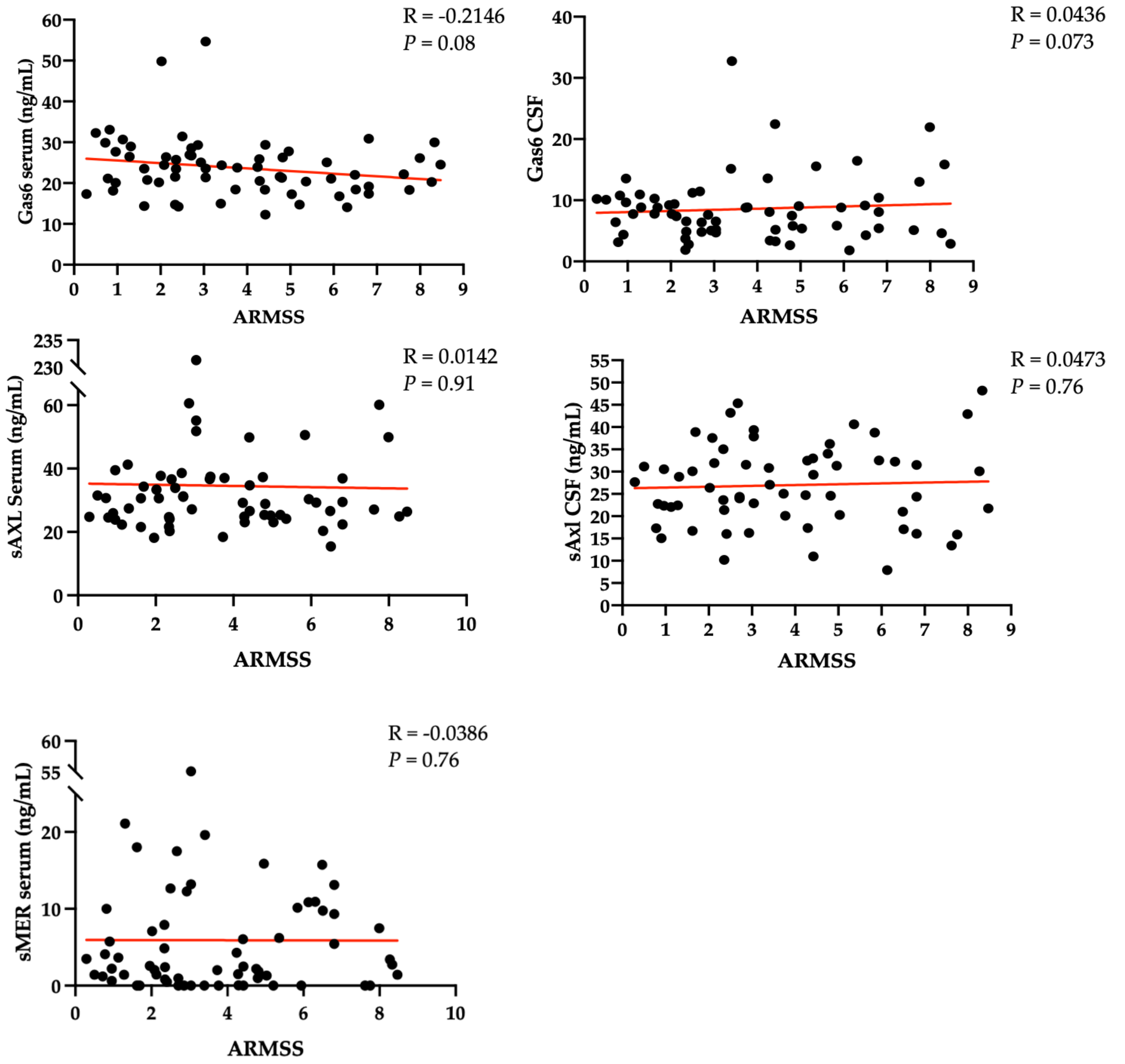

Figure 2. Spearman's correlation between Gas6 and TAM receptors in serum and CSF ARMSS. R = coefficient of correlation, *P* = *p*-Value

Table 8. We performed multivariate analysis the different types of MS.

Predictor	Coefficient	Standard error	*p* - value	95% confidence interval
Gas6 serum (ng/ml)	-0.00004	0.0053	0.99	-0.0106 – -0.0105
sAXL serum (ng/ml)	-0.0029	0.0029	0.32	-0.0090 – 0.0030
sMer serum (ng/ml)	0.0075	0.0054	0.16	-0.0033 – 0.0184
sTyro- 3 serum (ng/mL)	0.0157	0.0208	0.45	-0.0261 – 0.0576
NFL serum (pg/mL)	0.00005	0.0008	0.95	-0.0016 – 0.0017
Age	0.0009	0.0032	0.77	-0.0055 – 0.0073
Sex	-0.0424	0.0663	0.52	-0.1757 – 0.0907
N° brain lesion >10	-0.0517	0.0628	0.41	-0.1780 – 0.0745
N° spinal lesion >10	-0.0415	0.0656	0.53	-0.1735 – 0.0905
Gadolinium-enhancing	-0.0595	0.0638	0.35	-0.1878 – 0.0687

Table 9. We performed multivariate analysis for DMTs first line.

Predictor	Coefficient	Standard error	*p* - value	95% confidence interval
Gas6 serum (ng/ml)	-0.0008	0.0125	0.94	-0.0260 – 0.0243
sAXL serum (ng/ml)	-0.0087	0.0070	0.22	-0.0230 – 0.0055
sMer serum (ng/ml)	0.0158	0.0128	0.22	-0.0099 – 0.0415
sTyro- 3 serum (ng/mL)	0.0153	0.0493	0.75	-0.0837 – 0.1145
NFL serum (pg/mL)	0.0037	0.0019	0.059	-0.0001 – 0.0077
Age	-0.0177	0.0076	**0.024**	-0.0329 – -0.0024
Sex	-0.0331	0.1569	0.83	-0.3484 – 0.2822
N° brain lesion >10	0.3474	0.1487	**0.024**	0.0485 – 0.6463
N° spinal lesion >10	0.2401	0.1554	0.12	-0.0723 – 0.5524
Gadolinium-enhancing	0.0557	0.1511	0.71	-0.2478 – 0.3594
